# Comparison of high‐power and conventional‐power radiofrequency energy deliveries in pulmonary vein isolation using unipolar signal modification as a local endpoint

**DOI:** 10.1111/jce.14532

**Published:** 2020-05-15

**Authors:** Koichiro Ejima, Satoshi Higuchi, Kyoichiro Yazaki, Shohei Kataoka, Daigo Yagishita, Miwa Kanai, Morio Shoda, Nobuhisa Hagiwara

**Affiliations:** ^1^ Department of Cardiology Tokyo Women's Medical University Tokyo Japan; ^2^ Clinical Research Division for Heart Rhythm Management Tokyo Women's Medical University Tokyo Japan

**Keywords:** atrial fibrillation, human, outcomes, pulmonary vein isolation, radiofrequency ablation

## Abstract

**Introduction:**

Negative component abolition of the unipolar signal (unipolar signal modification [USM]) reflects the lesion transmurality. The purpose of this study was to compare the procedural safety and outcome between high‐power and conventional‐power atrial radiofrequency applications during a pulmonary vein isolation (PVI) using USM as a local endpoint.

**Methods and Results:**

High‐power (50 W) and conventional‐power (25‐40 W) applications were compared among 120 consecutive patients with paroxysmal atrial fibrillation who underwent a USM‐guided PVI. The first 60 patients were treated with conventional‐power (CP) group and last 60 with high‐power (HP) group. The atrial radiofrequency applications lasted for 5 to 10 seconds (CP group) or 3 to 5 seconds (HP group) after the USM. All procedures were performed using 3D mapping systems with image integration and esophageal temperature monitoring. The baseline characteristics were similar between the two groups. The HP group had fewer acute PV reconnections (62% vs 78%; *P* = .046) and a reduced procedure time (119.3 ± 28.1 vs 140.1 ± 51.2 minutes; *P* = .04). Freedom from recurrence after a single ablation procedure without any antiarrhythmic drugs was higher in the HP group than CP group (88.3% vs 73.3% at 12‐months after the procedure, log‐rank; *P* = .0423). There were no major complications that required any intervention.

**Conclusions:**

The high‐power PVI guided by USM decreased the procedural time and may improve the procedural outcomes without compromising the safety.

## 1 INTRODUCTION

Pulmonary vein isolation (PVI) is the cornerstone of atrial fibrillation (AF) ablation.^1^ The major cause of recurrent AF after the PVI is a reconnection of the PVs (PVR).^2^, ^3^ To reduce the recurrence rate after AF ablation procedures, the establishment of a dependable ablation strategy that enhances the quality of the radiofrequency (RF) applications during the PVI and achieves a so called durable PVI is desirable.

It has been demonstrated in a porcine model that complete elimination of the negative component of the unipolar atrial electrogram (R morphology achievement), while applying RF energy, is always associated with transmural lesions.^4^ R morphology achievement, the so‐called the unipolar signal modification (USM), has been reported as a useful guide for the endpoint of each RF application and provides a real‐time evaluation of the transmural lesion creation while performing the PVI.^5^, ^6^, ^7^

During RF ablation, a lesion is created by resistive heating followed by conductive heating. Resistive heating immediately causes irreversible myocardial tissue injury with cellular death, whereas conductive heating passively extends to deeper tissue layers causing potential reversible tissue damage. Recently published data has shown that a protocol with a high‐power RF delivery and shorter duration (HPSD) might lead to a beneficial shift involving an increased resistive heating and decreased conductive heating, which outweighs using a conventional‐power control with 30 to 40 W.^4^, ^8^

We hypothesized that the high‐power RF application under guidance of USM may enhance the durability of the isolated PV and procedural outcome of the AF ablation in patients with paroxysmal AF (PAF). The aim of this study was to compare the procedural safety and outcomes of high‐power and conventional RF power energy deliveries during the PVI using USM as a local endpoint.

## 2 METHODS

In this prospective cohort study, we enrolled 120 consecutive patients who underwent a first catheter ablation of PAF between April 2018 and July 2019 at our institution. PAF was defined according to the current guidelines.^1^ Patients who had structural heart disease and systolic dysfunction beyond the degree of moderate (left ventricular ejection fraction <40%) were excluded. All patients underwent a circumferential PVI (CPVI) guided by USM. The power output of the RF applications was 25 to 40 W in the first half of the patients (conventional‐power [CP] group, n = 60) and 50 W in the second half (high‐power [HP] group, n = 60). The study protocol conformed to the ethical guidelines of the 1975 Declaration of Helsinki. It was approved by the Institutional Review Board and Ethical Committee of the Tokyo Women's Medical University. Written informed consent was obtained from all patients.

The details of the peri‐procedural management and ablation technique have been described previously.^9^ As a preoperative evaluation, all patients underwent transthoracic echocardiography and multi‐detector computed tomography. Before the procedure, all antiarrhythmic drugs (AADs) were discontinued for at least five half‐lives except for amiodarone. All ablation procedures were performed by operators with experience of >200 AF ablation cases under conscious sedation and uninterrupted anticoagulants.

We performed a CPVI using a point‐by‐point ablation technique guided by electroanatomical mapping (CARTO) combined with image integration and using USM as a guide for the endpoint of each RF application.^9^ A 3.5 mm cooled‐tip catheter (Navistar ThermoCool SmartTouch SF, Biosense‐Webster Inc, Irvine, CA) was utilized for mapping and ablation. The irrigation flow rate was 8 mL/min and temperature limit 42℃. The targeted inter‐lesion distance was <5 mm. In both groups, all procedures were started during sinus rhythm.

**Figure 1:**
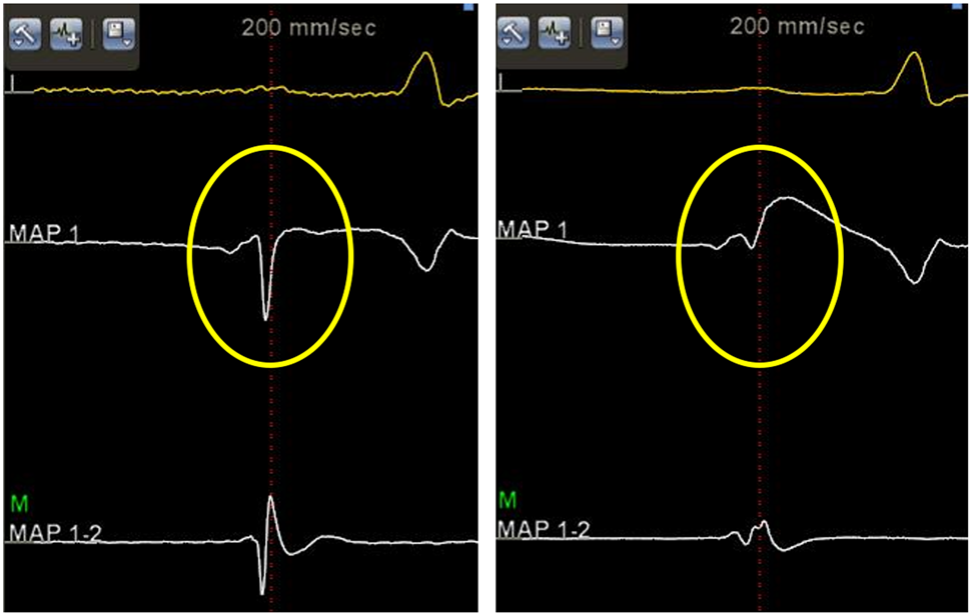
Unipolar atrial electrograms (EGMs) recorded before and after the ablation. This figure shows the intracardiac EMGs before (left panel) and after (right panel) the radiofrequency (RF) application. Surface electrocardiogram (ECG) lead I, unipolar EGMs (MAP 1), and bipolar EGMs (MAP 1‐2) recorded by the ablation catheter are shown. The atrial unipolar change from a positive‐negative morphology to exclusively a positive morphology (yellow circle)

In the CP group, the power output was 30 to 40 W, but up to 25 W at neighboring regions of the esophagus. Every single RF delivery was performed with a 10 to 20 g contact force (CF) and was continued for at least 5 seconds (5‐10 seconds) after the unipolar atrial electrogram that was recorded by the ablation catheter, which always demonstrated a positive‐negative morphology before the ablation, had become completely positive (USM; Figure 1).^6^

In the HP group, the power output was 50 W. Every single RF delivery was performed with a 5 to 20 g CF (target 10 g) and was continued for 3 to 5 seconds after the USM for the segments other than those adjacent to the esophagus. When the CF was >15 g or the ablation catheter was nearly perpendicular to the atrial wall, the RF delivery was continued for only 3 seconds after the USM. When the CF was <10 g or the RF application site was near the carina region, the RF delivery was continued for 5 seconds after the USM. The RF time was limited to ≤5 seconds and the CF was restricted to <10 g at neighboring regions of the esophagus. In both groups, the RF delivery was stopped in the case of chest pain or an esophageal temperature rise of >39℃ during the posterior wall ablation.

During each RF application, the USM was monitored in real‐time with the CARTO system at a sweep speed of 200 mm/s. A reference annotation signal was recorded from the bipolar signals of the electrode catheter placed in the coronary sinus. Unipolar signals were recorded from the 3.5 mm distal electrode of the ablation catheter and were filtered by the CARTO system with a 0.5 to 120 Hz band‐pass filter and displayed in the CARTO annotation viewer.^5^ The indifferent electrode was used as the cathode and was located at the level of the inferior vena cava.^6^

The settings of the automated ablation tag marking (VisiTag) were as follows: stability <2.5 mm for 3 seconds; CFmin was 25% of time ≥3 g; tag size was 2 mm; and a white‐colored tag was assigned when the ablation index (AI) was >320 and a red‐colored tag when the AI was >380. VisiTag was not utilized as a guide for the RF delivery but as one of the anatomical pieces of information of the potential site of a residual LA‐PV electrical connection after a circumferential RF application around the ipsilateral PVs.

A PVI was defined as bidirectional block between the LA and inside the CPVI area and was confirmed by the electrograms recorded from circular mapping catheters and pacing maneuvers followed by at least a 20‐minute waiting period. If an overt PVR did not occur, a bolus of intravenous adenosine (dose resulting in atrioventricular block, up to 60 mg) was administered to unmask any sites of dormant conduction after an intravenous injection of a 10 μg bolus of isoproterenol. Further ablation was performed at any sites of overt or unmasked reconnections to achieve the PVI once again. We targeted the ablation of any atrial tachyarrhythmias (ATAs) induced by programmed electrical stimuli with or without an injection of a 10 μg bolus of isoproterenol.

Ineffective AADs before the ablation were prescribed only if any early recurrences of an ATAs were observed before discharge and were discontinued by 3 months after the procedure. All patients were scheduled for visits in the outpatient clinic at 1, 3, 6, 9, and 12 months after the procedure and then every 6‐months. The presence of ATAs was evaluated by electrocardiographic recordings and 24‐hour ambulatory monitoring (3, 6, 9, and 12 months after the ablation and then every 6‐months). Patients with palpitations were encouraged to use portable electrocardiographic monitoring (HCG‐801R; Omron, Kyoto, Japan). Recurrence was defined as any episode of ATAs lasting >30 seconds after a 2‐month blanking period from the ablation procedure without any AADs. The anticoagulant treatment was discontinued at 3 months after the procedure unless the patients had a CHADS_2_ score of ≥2.

Data are presented as the mean ± standard deviation, percentage, or number, as appropriate. Differences between the continuous values were assessed using an unpaired two‐tailed *t* test for normally distributed continuous variables, Mann‐Whitney *U* test for skewed variables, and the *χ* ^2^ test (with Fisher's exact test) for nominal variables. The time‐to‐arrhythmia recurrence was estimated using the Kaplan‐Meier method and compared using the log‐rank test. A *P* < .05 was considered statistically significant. All statistical analyses were performed with JMP Pro software version 14.0.0 (SAS Institute, Cary, NC).

## 3 RESULTS

### 3.1 Patient clinical characteristics

The baseline characteristics of the patients are shown in Table 1. There were no significant differences in the baseline characteristics between the two groups.

**Table 1:** Baseline characteristics

	High‐power group (n = 60)	Conventional‐power group (n = 60)	*P* value
Age, y	63.0 ± 11.3	66.7 ± 8.9	.09
Male	44 (73)	42 (70)	.69
Hypertension	29 (48)	30 (50)	.86
Diabetes mellitus	10 (17)	12 (20)	.64
Previous TIA/stroke	6 (10)	7 (12)	.77
Sleep apnea	1 (2)	3 (5)	.31
eGFR, mL/min/1.73m^2^	65.4 ± 16.2	62.0 ± 18.2	.21
Body mass index, kg/m^2^	23.9 ± 2.8	23.8 ± 3.2	.96
CHA_2_DS_2_‐VASc score	1.8 ± 1.4	2.2 ± 1.4	.11
Left ventricular ejection fraction (%)	57.7 ± 3.9	57.4 ± 6.3	.88
E/e′	9.6 ± 3.1	10.2 ± 4.8	.85
Left atrial volume index, mL/m^2^	34.3 ± 10.3	36.1 ± 8.7	.23
Left common PV	5 (8)	3 (5)	.36
Right middle PV	3 (5)	1 (2)	.31

*Note*: All values are the mean ± SD or number (%).

Abbreviations: e′, early diastolic mitral annular velocity; E, early diastolic transmitral flow velocity; eGFR, estimated glomerular filtration rate; ​​​​​​PV, pulmonary vein; TIA, transient ischemic attack.

### 3.2 Procedural results

A CPVI was successfully performed in all patients. The other results of the first ablation procedure are shown in Table 2.

**Table 2:** Index AF ablation procedures

	High‐power group (n = 60)	Conventional‐power group (n = 60)	*P* value
Procedural time, min	119.3 ± 28.1	140.1 ± 51.2	.04
Fluoroscopic time, min	0.4 (0, 7.7)	10 (7, 14)	.0001
RF time, min	17.9 ± 7.2	34.9 ± 12.7	<.0001
Delivered energy, kJ	43.1 ± 17.3	65.9 ± 25.4	<.0001
Ablation time per point during the PVI, s	9.1 ± 1.1	25.0 ± 2.5	<.0001
Time for the PVI, min	25.9 ± 11.4	37.5 ± 20.1	.0001
Time for the left PVI, min	14.4 ± 8.6	24.2 ± 14.6	<.0001
Time for the right PVI, min	11.5 ± 7.2	14.3 ± 9.9	.044
Patients with time‐dependent acute PV reconnections	30 (50)	39 (65)	.096
Left PV	22 (37)	30 (50)	.14
Right PV	18 (30)	22 (37)	.44
Ipsilateral	40/120 (33)	52/120 (43)	.13
Patients with ATP‐induced dormant conduction	14 (23)	24 (40)	.0497
Left PV	5 (8)	19 (32)	.001
Right PV	9 (15)	9 (15)	1.00
Ipsilateral	14/120 (12)	28/120 (23)	.03
Patients with acute PV reconnections	37 (62)	47 (78)	.046
Procedures other than a PVI			
SVC isolation	57 (95)	57 (95)	1.00
CTI linear ablation	18 (30)	18 (30)	1.00
Other ablation targets	4 (7)	4 (7)	1.00
Atrioventricular reentrant tachycardia	1	1	
Atrioventricular nodal reentrant tachycardia	1	0	
Focal atrial tachycardia	2	4^a^	
Complication	1 PNP	0	

*Note*: All values are the mean ± SD or number (%).

Abbreviations: AF, atrial fibrillation; ATP, adenosine triphosphate; CTI, cavo‐tricuspid isthmus; PNP, phrenic nerve palsy; PV, pulmonary vein; PVI pulmonary vein isolation; RF, radiofrequency; SVC, superior vena cava.

^a^ One patient had two atrial tachycardias.

In the HP group, the procedural time, fluoroscopic time, RF time, and ablation time per point during the PVI were significantly shorter and the delivered energy was significantly lower than that in the CP group. Even when it was limited to the patients who underwent only thoracic vein isolation (CPVI and superior vena cava isolation), the procedural time (108.9 ± 22.6 vs 123.6 ± 27.1 minutes; *P* = .03), fluoroscopic time (0.3 [interquartile range {IQR}: 0, 6] vs 9.5 [IQR: 7.0, 12.3] minutes; *P* < .0001), and RF time (15.6 ± 5.6 vs 31.6 ± 9.9 minutes; *P* < .0001) were significantly shorter and the delivered energy (37.9 ± 13.8 vs 58.4 ± 18.5 kJ; *P* < .0001) significantly lower in the HP group than CP group.

However, we attempted to perform a zero fluoroscopic AF ablation procedure in some patients in the HP group, and the difference in the fluoroscopic time between the two groups was not caused by the difference in the prespecified procedural strategies. Although the time‐dependent acute PVRs were comparable between the two groups, ATP‐induced dormant conduction was more frequently observed in the CP group than HP group. The number of patients with an acute PVR, both time‐dependent and ATP‐induced, was greater in the CP group than HP group.

### 3.3 Follow‐up and redo AF ablation procedures

**Figure 2:**
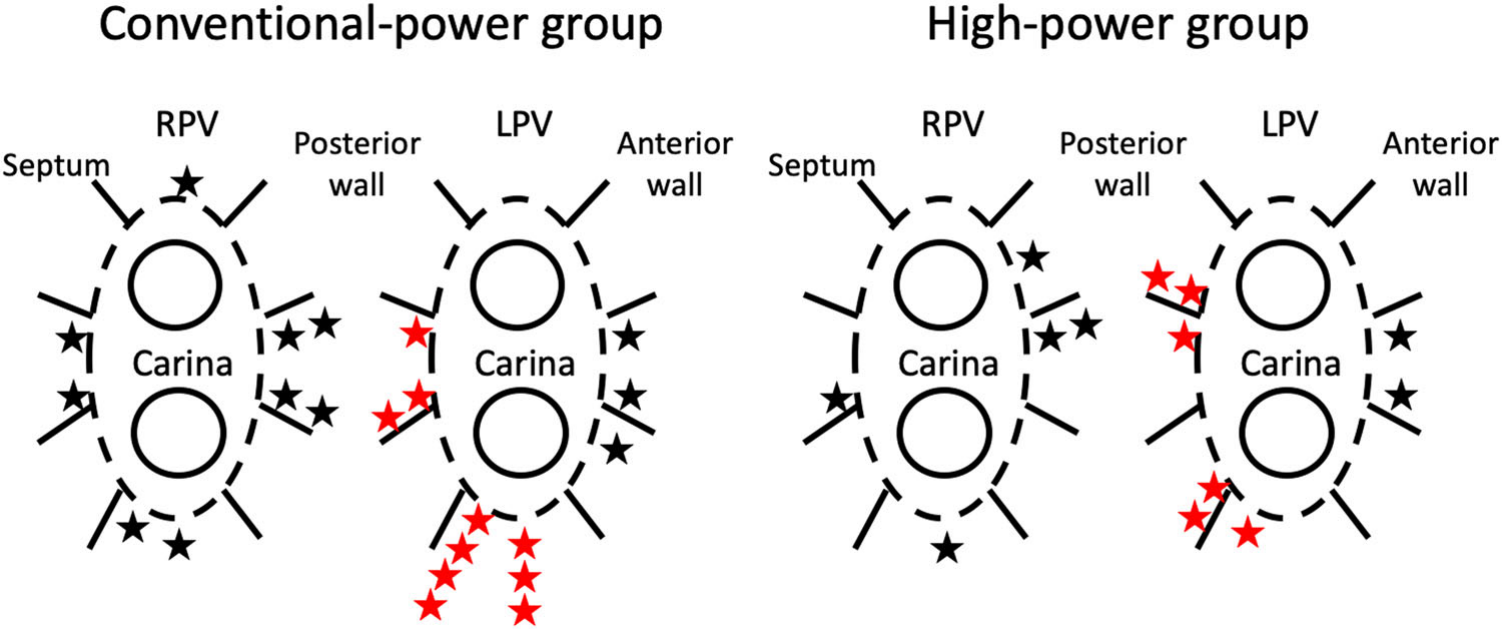
Spatial distribution of the sites of the reconnections during the redo procedure in both treatment groups. The stars indicate the site of the reconnections. The red stars indicate the site of the reconnections neighboring the esophagus. LPV, left pulmonary vein; RPV, right pulmonary vein ​​​​​

During a mean follow‐up of 20.7 ± 2.0 months after a single ablation procedure, 17 (28%) patients had an ATA recurrence in the CP group, including PAF in 15 patients and paroxysmal AT in 2. Out of those 17 patients, 13 underwent redo procedures and all of them had PVRs including right PVRs in 7 and left PVRs in 9. On the other hand, during a mean follow‐up of 12.5 ± 2.9 months after a single ablation procedure, seven (12%) patients had a PAF recurrence in the HP group. All seven of these patients underwent redo procedures and six of seven patients had PVRs including right PVRs in 4 and left PVRs in 5. The spatial distribution of the sites of the PVRs during the redo procedures in both groups is shown in Figure 2. In both groups, sites near the carina region and sites neighboring the esophagus were the major reconnection sites.

**Figure 3:**
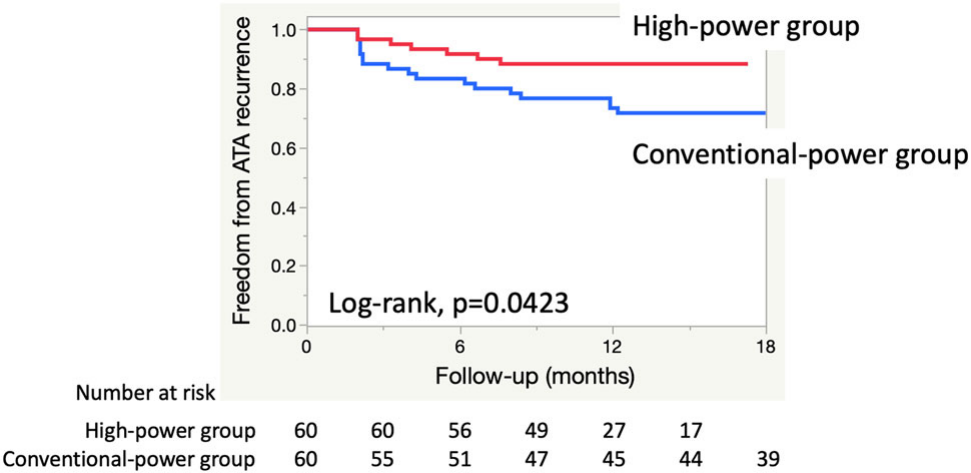
Kaplan‐Meier survival curves showing the freedom from atrial tachyarrhythmias after a single procedure without any antiarrhythmic drugs in both groups. ATA, atrial tachyarrhythmia

A Kaplan‐Meier survival analysis showed that the ATA recurrence‐free rate after a single ablation procedure without any AADs was higher in the HP group than CP group (log‐rank; *P* = .0423; Figure 3). The ATA recurrence free survival rate at 1 year after a single procedure was 88.3% in the HP group and 73.3% in the CP group, respectively.

Asymptomatic phrenic nerve injury occurred in one patient in the HP group. No gastric hypomotility, atrio‐esophageal fistulae, cardiac tamponades, or strokes occurred in either group.

## 4 DISCUSSION

The main finding of this study was that high‐power RF applications were associated with not only a significant reduction in the procedure time, RF time, delivered energy, and acute PVRs but also an improvement in the ATA recurrence free rate after a single ablation procedure as compared with conventional‐power applications in patients that underwent a PVI of PAF guided by USM.

Because the main cause of recurrence after AF ablation is a PVR, we should make special effort to achieve a durable PVI to improve the procedural outcome. During the initial ablation phase, the delivery of RF energy results in direct tissue heating during the resistive phase. This zone of direct heating serves as a heat source for passive heat diffusion into deeper tissue layers during the conductive phase. As the ablation continues, lesion expansion occurs predominantly by convective heating. The acute lesion is composed of tissue heated to a lethal temperature and tissue heated to a sublethal temperature with reversible injury.^8^ An HPSD RF application increases the effect of resistive heating with a high‐power and reduced conductive heating by a shorter energy delivery. Therefore, high‐power ablation may theoretically favor the creation of durable lesions. In the present study, high‐power RF applications reduced the procedural time, acute PVRs, and ATA recurrences during the follow‐up period.

Previous clinical studies have evaluated the HPSD ablation during the PVI procedure using several endpoints for each RF application, such as the RF time,^10^, ^11^, ^12^, ^13^, ^14^ CF‐related indices (ablation index^11^, ^15^, ^16^, ^17^ and lesion size index^11^, ^18^), and loss of pacing capture during the RF delivery.^18^ However, little is known about high‐power RF ablation for a PVI using USM as a guide for the local endpoint of each RF application.^19^

The USM, which is the complete elimination of the negative component of the unipolar atrial electrogram (R morphology achievement), while applying RF energy, reflects a transmural lesion.^4^ USM is a qualitative indicator and indicates the real‐time evaluation of the transmural lesion creation while performing the PVI. It has been reported that the USM is a useful guide for the endpoint of each RF application while performing the PVI.^5^, ^6^, ^7^, ^19^

In some cases, however, the USM did not translate into transmural necrosis but instead into transmural lesions only (with the potential for reversibility), likely related to transient cell damage creation.^20^ To create transmural and irreversible (rather than functional and potentially reversible) lesions, extended RF applications for a few seconds after the negative component of the USM are needed.^20^

Pambrun et al^19^ conducted a comparison of the high‐power (40‐50 W) and standard‐power (25‐30 W) PVI guided by USM under general anesthesia in 100 patients with PAF. In the high‐power group, each RF application was stopped 2 seconds after the USM based on their animal study, which achieved a transmural lesion in 95% of subjects. An acute PVR after a 20‐minute waiting time without an adenosine challenge was observed in only 2% of ipsilateral PVs. The sinus rhythm maintenance rate 1 year after a single ablation procedure was 90% without AADs, which was similar to that of the standard‐power group.

In the present study, we compared the procedural safety and outcome of high‐power (50 W) and conventional‐power (25‐40 W) RF energy deliveries in the PVI using USM as a local endpoint under conscious sedation in 120 patients with PAF. The ablation strategy in the conventional‐power group was the same as in the COMPASS study.^6^ In the high‐power group, each RF application was extended for 3 to 5 seconds after the USM depending on the CF, RF application site, and catheter tip orientation. Time‐dependent (with at least a 20 minute waiting time) ATP‐provoked acute PVRs were observed in 62% of the patients. One year after a single ablation procedure, 88% of the patients in the high‐power group were free from ATAs without AADs, which was higher than that in the conventional‐power group.

In the high‐power group in the present study, RF applications, except at sites neighboring the esophagus, were applied with 50 W for around 10 seconds with a target CF of 10 g, which corresponded to an AI value of 400.^21^ An AI of 400 au was equal to the target AI value on the posterior wall and less than the target AI value on the anterior wall in the previously reported AI‐guided PVI using a power setting of 35 to 45 W.^17^, ^22^ On the other hand, the RF applications at sites neighboring the esophagus were 50 W for ≤5 seconds with a target CF of <10 g, which corresponded to an AI value of <300.^21^ The lesion depth has been reported to be significantly smaller with an increasing RF power and shorter RF duration while using the same target AI in ex vivo animal models.^21^ This lesion characteristic is favorable for avoiding collateral tissue damage. A study evaluating the incidence of esophageal thermal injury assessed by late gadolinium enhancement magnetic resonance imaging in HPSD RF applications (50 W for 5 seconds) was shown to be similar to that with low‐power long‐duration RF applications (≤35 W for 10‐30 seconds).^23^ A large observational study of almost 14 000 AF ablation procedures exhibited an extremely low complication rate of the HPSD ablation strategy (45‐50 W for 2‐15 seconds).^24^ In the present study, no PVI related severe complications occurred.

We performed the ATP challenge after a waiting time. Because many patients underwent ablation other than a PVI, the waiting time was relatively long in our study. It may be one reason for the relatively high rate of an acute PVR in the high‐power group in our study as compared with the previous study.^19^ The difference in the anesthesia strategies may be another reason for the high acute PVR rate in our study.

We evaluated the PVR site during the redo procedures in both groups. In both groups, the site near the carina region and sites neighboring the esophagus were the major reconnection sites. While further effort to improve the durability of the PVI is necessary, it may need an unavoidable trade‐off between the effectiveness and procedural safety.

CF and some CF‐related indices (force‐time integral, ablation index, and lesion size index) are known to be well correlated with the lesion size when using RF current for catheter ablation.^25^ Those are the indicators of the RF application, which provide uniformity regardless of the thickness of the target lesion. On the other hand, the USM‐guided ablation is a tailored approach for each target lesion and can be used with whatever ablation catheter you wish without any extra cost. A high‐power PVI guided by USM is an ideal ablation strategy in terms of shortening the procedural time, favorable procedural outcomes, and the safety.

There were several limitations to the present study. First, this was a single center and nonrandomized study with a limited number of patients. Our findings are preliminary and are not adequate to prove our hypothesis and should be confirmed by a randomized study with a larger sample size and longer follow‐up. Second, the patient population of this study was limited to patients without structural heart disease or systolic dysfunction. Third, the recurrence rate of ATAs may have been underestimated because asymptomatic ATA episodes may have been undetected and our monitoring using 24‐h ambulatory monitoring could detect less ATA episodes than other longer monitoring methods using 7‐day ambulatory monitoring or implantable loop recorders.

## 5 CONCLUSION

The ablation strategy with a PVI based on a high‐power RF application guided by USM shortened the procedural time and improved the procedural outcome without compromising the safety profile.
